# No immediate attentional bias towards or choice bias for male secondary sexual characteristics in Bornean orang-utans (*Pongo pygmaeus*)

**DOI:** 10.1038/s41598-024-62187-9

**Published:** 2024-05-27

**Authors:** Tom S. Roth, Iliana Samara, Juan Olvido Perea-Garcia, Mariska E. Kret

**Affiliations:** 1 Cognitive Psychology Unit, Institute of Psychology, The Faculty of Social and Behavioral Sciences, Leiden University, Wassenaarseweg 52, 2333 AK Leiden, The Netherlands; 2 Apenheul Primate Park, J.CWilslaan 21, 7313 HK Apeldoorn, The Netherlands; 3 Animal Behaviour & Cognition, Department of Biology, Utrecht University, Padualaan 8, 3584 CH Utrecht, The Netherlands; 4 Leiden Institute of Brain and Cognition (LIBC), Leiden, The Netherlands

**Keywords:** Animal behaviour, Sexual selection

## Abstract

Primate faces provide information about a range of variant and invariant traits, including some that are relevant for mate choice. For example, faces of males may convey information about their health or genetic quality through symmetry or facial masculinity. Because perceiving and processing such information may have bearing on the reproductive success of an individual, cognitive systems are expected to be sensitive to facial cues of mate quality. However, few studies have investigated this topic in non-human primate species. Orang-utans are an interesting species to test mate-relevant cognitive biases, because they are characterised by male bimaturism: some adult males are fully developed and bear conspicuous flanges on the side of their face, while other males look relatively similar to females. Here, we describe two non-invasive computerised experiments with Bornean orang-utans (*Pongo pygmaeus*), testing (i) immediate attention towards large flanges and symmetrical faces using a dot-probe task (*N* = 3 individuals; 2F) and (ii) choice bias for pictures of flanged males over unflanged males using a preference test (*N* = 6 individuals; 4F). In contrast with our expectations, we found no immediate attentional bias towards either large flanges or symmetrical faces. In addition, individuals did not show a choice bias for stimuli of flanged males. We did find exploratory evidence for a colour bias and energy efficiency trade-offs in the preference task. We discuss our null results and exploratory results in the context of the evolutionary history of Bornean orang-utans, and provide suggestions for a more biocentric approach to the study of orang-utan cognition.

## Introduction

Primates have a highly specialized visual system that helps them navigate their social environment^1,2^. For example, primates attend to faces of conspecifics^3^ and discriminate faces based on different characteristics, such as emotional expressions^4^ and familiarity^5–7^. Importantly, primate faces can also signal cues that are relevant for mate choice, such as health or dominance^8^. Consequently, primates might have cognitive biases related to sexually relevant facial characteristics. Examples of such traits are sexually dimorphic characteristics and facial symmetry^9,10^. However, previous work on this topic has mostly focused on rhesus macaques, and very few studies have studied cognitive biases to sexually relevant facial characteristics in great apes. Therefore, the present paper aims to investigate whether Bornean orang-utans (*Pongo pygmaeus*) have cognitive biases for such facial characteristics using an immediate attention task and a preference task.

Cognitive processes are strongly influenced by evolutionarily relevant contexts, such as mate choice^11,12^. Mate choice is one of the most important aspects of a sexually reproducing animal’s life: choosing a suitable mate might ensure a good representation of the individual’s genes in the next generation. Because of this strong incentive to choose a suitable mate, many species have evolved specific mate preferences that guide individuals during the mate choice process ^13,14^. For humans, it has been established that preferences affect social cognition: several cognitive processes, such as attention^15,16^, memory^17^, and motivational drive^18^, are modulated by physical attractiveness. For non-human primates, research on this topic is still relatively scarce. While previous work has mainly focused on looking preferences on a longer timescale (multiple seconds), and how these are modulated by sexual dimorphic traits^19–21^, few studies have investigated other cognitive processes such as immediate attention or choice bias^22^. Furthermore, the studies that investigated this topic are almost exclusively restricted to rhesus macaques (*Macaca mulatta*), although a good understanding of the cognitive processes associated with sexual selection requires investigation of a wide range of species instead of focusing mostly on one species.

Attentional biases towards specific facial characteristics have been found in multiple studies with rhesus macaques. Seminal work by Waitt and colleagues showed that rhesus macaque (*Macaca mulatta*) females have an attentional bias towards bright red male faces when they were paired with paler male faces^21^, while males seemed to prefer bright red female hindquarters, but not faces ^23^. Similarly, macaques seem to be biased towards symmetrical faces ^24^. Later studies with free-ranging macaques on the island of Cayo Santiago have extended these previous findings by showing attentional biases towards facial photographs of ovulating within-group females in males^25^, while females seem to preferentially attend to more masculine male faces^20^. Furthermore, individuals of both sexes looked longer at dark red male faces than pink male faces, suggesting that red coloration plays a role in both female choice and male competition^19^. In short, these studies collectively provide strong evidence for the notion that macaques selectively attend to specific facial characteristics that are relevant for mate choice.

Orang-utans are a suitable species to study cognitive biases associated with sexual selection. Unique among mammals, orang-utans are characterised by male bimaturism: while some adult males quickly develop secondary sexual characteristics, such as a throat sac, large body size, and conspicuous flanges on the sides of the face, other males experience developmental arrest of these characteristics^26^. These so-called unflanged males are sexually mature and can successfully reproduce, although females prefer to mate with fully developed flanged males when they are fertile^27^. Possibly, female preference for flanged males reflects selection for good genes: the transition from unflanged to flanged male is energetically costly, meaning that males of higher genetic quality would be more likely to develop into flanged males^27^. Furthermore, fierce male-male competition has been described between flanged males, suggesting that flanged males are also at serious risk of being injured during fights^28^. Thus, by mating with flanged males, orang-utan females might ensure that their offspring has higher genetic quality. Consequently, it could be beneficial to have cognitive biases to flanged males.

It important to note that the distinction between unflanged and flanged males is not as binary as it might appear at first glance. Unflanged males will eventually develop into fully flanged males^28^. During this transition, they will slowly start growing a larger body and might already show some early flange growth^29^. At the end of this transition period, the flanges will grow to their maximum size in a relatively short amount of time (~ 1 year, but varying between individuals^28^). Therefore, there might be several males that have started developing flanges but cannot yet be considered fully flanged. Furthermore, Knott (2009) proposes an additional distinction between past-prime flanged males and prime flanged males^30^. Past-prime flanged males are characterized by reduced flange size and seem to be less preferred by females. In short, while orang-utan males can be broadly categorized in flanged and unflanged males, it is important to realize that there are males with small or intermediate-size flanges because they are either still growing them or are past their prime.

Another trait that is often mentioned with regard to mate choice is facial symmetry, which might reflect the ability to withstand environmental stress during development^31^. Accordingly, humans seem to be more attracted to symmetrical faces than asymmetrical faces^10^, although recent studies are less conclusive^32^. While clear associations between health and facial symmetry have not been established in humans yet^33^, previous work on chimpanzees (*Pan troglodytes*)^29^ and rhesus macaques^35^ has found that more asymmetrical individuals were also less healthy. In addition, rhesus macaques prefer to look at symmetrical faces of conspecifics^24^, which shows that this facial characteristic might modulate attentional processes. Thus, individuals might have cognitive biases for symmetrical conspecifics, because selecting a mate with a symmetrical face could potentially result in more genetically fit offspring.

In this study, we employ two paradigms to investigate immediate attentional bias towards flanged faces and symmetrical faces, and choice bias for flanged faces. In the dot-probe task^36,37^, two different stimuli are simultaneously displayed, each one on a different side of the screen. After a set amount of time, both pictures disappear, and a dot appears on the location of one of the pictures. If the dot appears behind the stimulus that the participant was attending to, the participant can quickly indicate the location of the dot by clicking it. However, if the dot appears behind the stimulus that was not receiving the participant’s attention, they will first need to switch their attention to the dot before they can indicate the location. Thus, if the dot appears behind a stimulus that immediately attracts attention, participants will be faster to respond than when the dot appears behind a less salient stimulus^37^. Recently, the dot-probe task has been used to study emotion perception in different primate species^34–38^. In addition, the task has successfully been used in humans to study attractiveness bias^15,43,44^. In general, these studies have established that individuals immediately attend to evolutionarily relevant stimuli, such as emotional expressions or preferred partners. Therefore, we here employed the dot-probe task to study direct attention towards sexually relevant facial characteristics in orang-utans.

When it comes to choice bias, Watson et al.^22^ developed a paradigm to test choice biases in unrestrained primates. In this task, individuals first learn to associate two coloured dots (red and green) with specific categories (e.g., pictures of faces), so that they can predict what they will see on the screen by clicking a specific dot. During the test phase they can choose between the two coloured dots: both choices yield the same reward, but the picture that will appear on the screen is different. The authors successfully used this method to study preference for sex and status in rhesus macaques: they found that rhesus macaques chose to look more at faces of dominant males and perinea of conspecifics, while they were less likely to choose pictures of low-ranking conspecifics. Because this task has been successfully applied to rhesus macaques, here we used an adapted version to study choice bias for flanges in orang-utans.

The present paper reports the results of two studies. These two studies aimed to investigate immediate attentional biases towards flanged and symmetrical faces, and a choice bias for flanged faces in Bornean orang-utans, respectively. Given that the presence of flanges or facial symmetry may be a signal of good genes, we predicted for the dot-probe task that individuals should respond faster on trials where the dot would replace stimuli that depicted males with large flanges or males with symmetrical faces than when the dot replaced stimuli that depicted males with small or no flanges or asymmetrical faces. For the choice task, we expected individuals to more often choose the coloured dot that was associated with pictures of flanged males over the coloured dot that was associated with unflanged males.

Furthermore, for the preference task, we retrospectively decided to explore (i) whether individuals had a colour bias, (ii) whether individuals made choices that might reflect conservation of energy, and (iii) whether individuals showed temporal clustering in their choices, i.e., whether individuals switch between selecting flanged and unflanged stimuli every other trial or whether their choices are more clustered (e.g., multiple choices for one type of stimuli in a row). We investigated colour bias because evolutionary theories of colour vision have suggested that the ability to see red co-evolved with frugivory^45^. With regard to energy conservation, Bornean orang-utans are characterised by extremely low rates of energy use^46^, potentially an adaptation to habitats with long periods of fruit scarcity resulting in negative energy balance^47,48^. Potentially, such energy conservation mechanisms could also influence their responses during the task. Lastly, we also investigated temporal clustering, because flanged males are not only preferred mating partners^27^, but might also pose a threat (e.g., risk of infanticide^23^) or are perceived as threatening^49^. Consequently, individuals may show temporal clustering in their choices during our task, by either opting for a less arousing picture of an unflanged male after seeing a flanged male stimulus (i.e., more switching, temporal dispersion) or by mostly sampling flanged male stimuli, until arousal reaches a certain threshold and individuals switch to unflanged stimuli instead (i.e., fewer switches, temporal clustering). Thus, because it could provide an opportunity to learn more about the relationship between orangutan’s socio-ecology and their cognitive biases, we decided to explore these three topics in addition to our main questions.

## Methods

### Subjects and housing

The animals that participated in this study were part of a population of 9 Bornean orang-utans (*Pongo pygmaeus*) at Apenheul Primate Park, The Netherlands (Table 1). They were kept in a fission–fusion housing system consisting of 4 enclosures, meaning that they were in small subgroups with changing composition over time, in order to mimic the natural social system of the species. Some individuals never shared enclosures to avoid conflict (e.g., the two adult males). Each enclosure consisted of an inside part and an outside part. The orang-utans were fed multiple times a day, and had ad libitum access to water. Most of the orang-utans had previously been exposed to touchscreens for a previous dot-probe study^38^, but only two of those orang-utans (Sandy & Samboja) eventually participated in this dot-probe study.

**Table 1:** Orang-utans housed in Apenheul at the time of study.

Name	Sex	Date of birth	Origin	Dot-probe	Preference test
Kevin	M	~ 1982	Wild		
Sandy	F	29-4-1982	Captive	Yes	Yes
Wattana	F	17-11-1995	Captive		Yes
Amos	M	20-12-2000	Captive		
Samboja	F	9-6-2005	Captive	Yes	Yes
Kawan	M	22-2-2010	Captive	Yes	Yes
Baju	M	2-12-2015	Captive		Yes
Indah	F	19-10-2017	Captive		Yes

With regard to participation in the experiments described here, three individuals participated in the dot-probe experiments (both flange size and symmetry version), while six individuals participated in the preference test. Table 1 indicated which individuals participated in the experiments.

### Apparatus

Touchscreen experiments were conducted via E-Prime 2.0 on a TFT-19-OF1 Infrared touchscreen (19″, 1280 × 1024 pixels). The touchscreen setup was encased in a custom-made setup which was incorporated in one of the orang-utans’ night enclosures. This night enclosure could be made accessible from two of the main enclosures by the animal caretakers. The researchers controlled the sessions on a desktop computer connected to the touchscreen setup and could keep track of the orangutans’ responses on the touchscreen through a monitor that duplicated the touchscreen view. Additionally, the researchers had access to a livestream with a camera that was built in the enclosure, allowing them to observe the participant. Correct responses were rewarded with a sunflower seed on a 100% fixed reinforcement ratio. For most individuals, the rewards were delivered by a custom-built autofeeder linked to the desktop computer, that dropped a reward in a PVC chute. However, Kawan and Baju did not habituate properly to the presence of the feeder, and kept trying to push it over with sticks. Therefore, we decided to reward them manually. The researcher was positioned behind the setup which prevented visual contact between the orangutans and researchers.

### Stimuli

#### Dot-probe task

For the dot-probe task with flange size manipulation, we collected 72 images depicting front-facing Bornean or Sumatran orang-utan males with flanges. The images were collected through image hosting websites and social media groups. Due to the origin of the pictures, and the often-lacking information about the depicted individuals, we cannot be entirely certain that some stimulus combinations depict the same individuals. Furthermore, we often could not find a clear mention of the species depicted, which is why we consider the stimulus set as a combination of Bornean and Sumatran orang-utan males. We expected the species depicted to have little to no influence on our results because (1) facial features of Sumatran and Bornean flanged orang-utans are relatively similar^50^, (2) orang-utans are known to hybridize in captivity^51^ and (3) each stimulus would serve as its own control (i.e., we would present two modified stimuli based on the same face) meaning that no combinations of Bornean and Sumatran orang-utans would be shown.

We edited the stimuli in GIMP (v2.10.32). First, we cropped the faces. Second, we consecutively selected the flanges on the left and right side of the face, respectively. We defined the width of the flange as the distance between the horizontally most peripheral point of the face and the most peripheral point of either the eye region or beard. Hereafter, we increased the width of the flanges (measured in pixels) with 15 percent to obtain the stimulus with enlarged flanges, and we decreased the width with 15 percent to obtain the stimulus with reduced flanges. We chose 15 percent to make sure that the stimuli would not become abnormal in terms of flange size. In total, this resulted in 72 combinations of enlarged and reduced stimuli.

Using the same 72 images, we created the stimulus set for the dot-probe with symmetry manipulation. Here, we could only include the images where the faces of the orang-utans appeared to be nearly exactly frontally facing. To determine this, we visually inspected whether the eyes and nostrils were at a similar distance from the vertical midline of the face and whether they were of approximately similar size. This was the case for 49 of the images. Next, we created symmetrical versions of the face by mirroring either the left or the right hemisphere at the vertical midline of the face. Thus, from every stimulus, we obtained two symmetrized versions: one based on the left hemisphere and one based on the right hemisphere. Importantly, in some stimuli we employed an extra step to remove cross-eyedness that resulted from the mirroring. To this effect, we selected one of the eyes, and mirrored it, resulting in more congruent gaze direction of the eyes. Furthermore, some of the mirrored stimuli were characterised by abnormal facial shape, which is a well-known issue in symmetrized stimuli ^10^. If this was the case, we excluded the stimulus. In total, we obtained 80 stimulus pairs consisting of one symmetrized face and the original face showing natural variation in symmetry.

#### Preference task

For the preference task, we used 104 stimuli (52 flanged, 52 unflanged) of Bornean orang-utans. The stimuli were collected from the Internet, mainly from release reports published by Bornean orang-utan reintroduction programs. These were supplemented with portrait pictures taken from semi-wild orang-utans and pictures of zoo-housed orang-utans within the orang-utan EEP. All of the stimuli depict front-facing Bornean orang-utan males. We cropped their faces using GIMP (v2.10.32) and pasted the cropped faces on a light-grey background (#808,080), resulting in stimuli with an 18:13 aspect ratio. From both the flanged and the unflanged stimuli, we randomly selected four stimuli (eight in total) to use as stimuli in the forced-trial phase of the experiment. The remaining 48 stimuli of each category were randomly distributed across three sessions.

### Procedure

#### Dot-probe task

The procedure for the dot-probe task was almost identical to the one described in Laméris et al.^38^. In five months prior to the experiment, all individuals were allowed to participate in training sessions. For training, we followed the protocol previously used to train bonobos (*Pan paniscus*) and Bornean orang-utans on the dot-probe task^38,52^. We elaborate on the different steps and the individual trajectories of the training period in the Supplementary Materials (Supplementary Methods & Supplementary Table 1). Eventually, three individuals fulfilled the training criteria. They participated in the full task.

**Figure 1:**
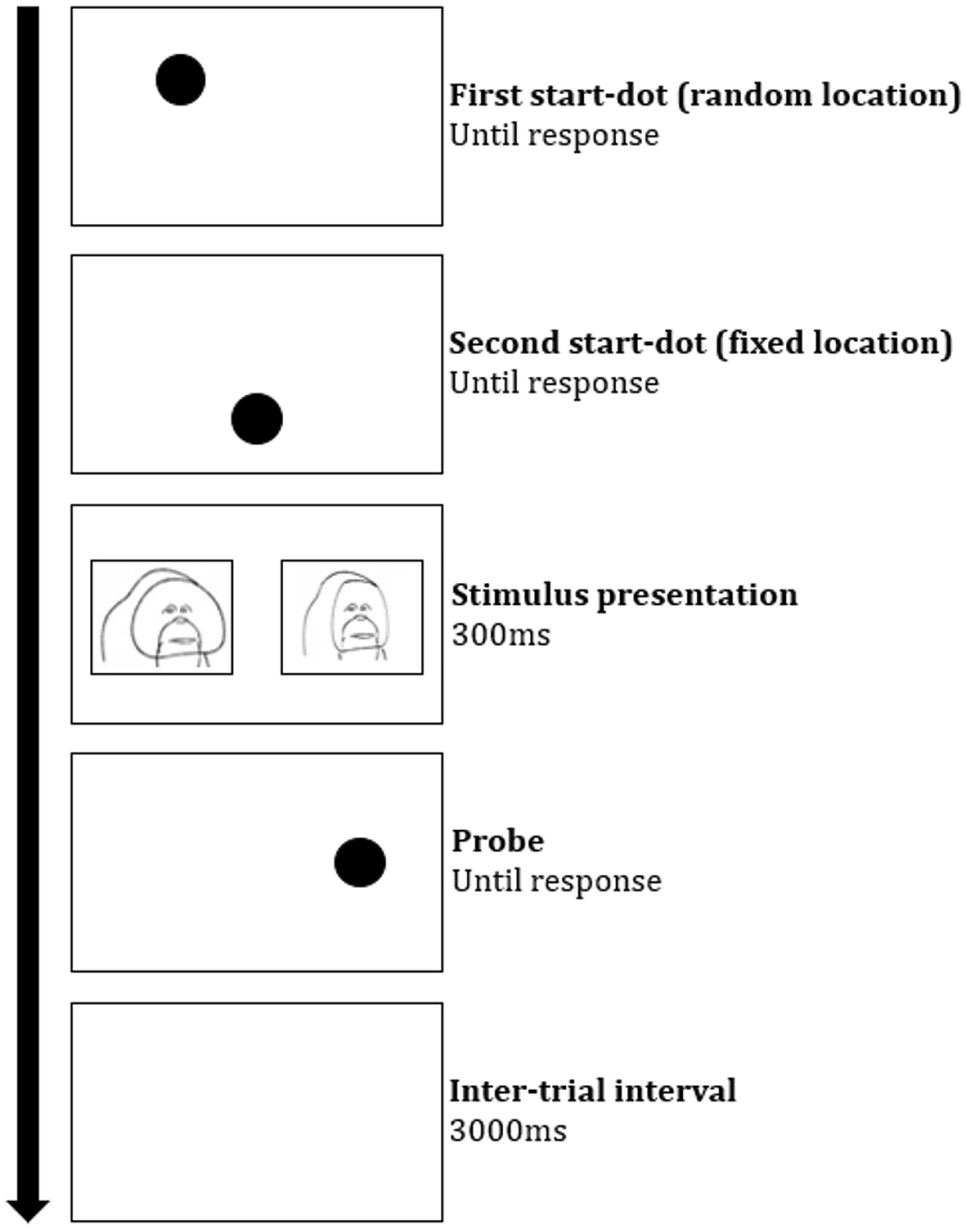
Schematic depiction of a dot-probe task trial with large and small flanges as competing stimuli. The arrow indicates the temporal progression of the trial.

Regarding the task design, a trial consisted of five phases (Fig. 1). First, a 200 × 200-pixel black dot appeared on a random position on the screen and had to be clicked. We added this step to avoid anticipatory responses. Second, the dot appeared in the lower, middle part of the screen. Touching this dot activated presentation of two stimuli (500 × 375 px) that were vertically positioned in the middle of the screen, and horizontally equidistant from the center of the screen (20% vs. 80%). After 300 ms, the stimuli disappeared and only one of the stimuli was replaced by a dot (the probe) that remained on the screen until touched by the subject. Touching the dot resulted in a reward (sunflower seed). After an inter-trial interval of 3 s, a new trial started. The background of screen was white during all steps of the trial.

Trials were presented in randomized order. For the flange size dot-probe, each individual participated in 6 sessions consisting of 24 trials. For the symmetry dot-probe, each individual participated in 8 sessions consisting of 20 trials. All stimuli were presented twice across all sessions: once as probed stimulus (replaced by dot), once as distractor stimulus (not replaced by dot). At the end of the test sessions, we created extra sessions per subject to repeat outlier trials (see Statistical analysis). All data were collected between February and December 2020, with a test stop between March and July 2020 due to COVID-19.

Two of the three participating individuals were already trained on the task for a previous study^38^. They received a few training sessions to check whether they still executed the task correctly, which was the case. For the other individuals, we employed a similar training procedure. Only one of the individuals, Kawan, managed to pass all phases of the training (between July and December 2019). Thus, this resulted in a total sample of three participants.

#### Preference task

**Figure 2:**
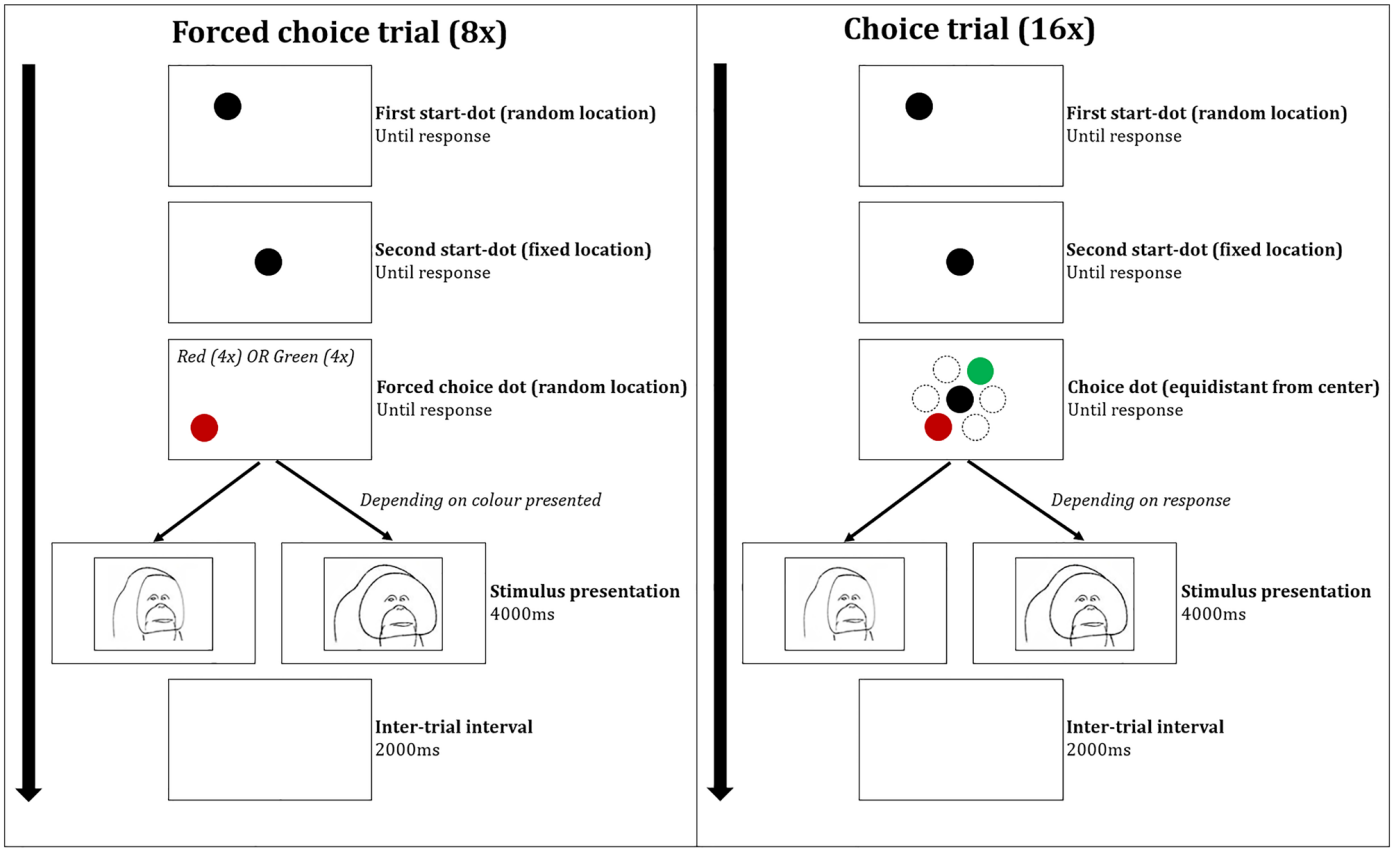
Schematic depiction of two preference task trials with flanged and unflanged stimuli. The left box shows the design of a forced choice trial, while the right box shows the design of a choice trial. The arrows indicate the temporal progression of the trial.

The procedure of the preference task was adapted from Watson et al.^22^. Each session consisted of two parts (Fig. 2): a forced-trial procedure (8 trials) and a choice-trial procedure (16 trials). During all parts of the experiment, the background was silver gray (#c0c0c0). Trials in the forced-trial procedure started with a 300 × 300-pixel black dot that appeared in a random position. This randomly located dot was added at the start of each trial to avoid anticipatory responses. After clicking the dot, a similar dot appeared exactly in the center of the screen. By clicking this dot, individuals would advance to a screen that depicted either a red dot or a green dot. The shades of green (#339,900) and red (#990,000) were almost equal in saturation. Each dot colour was associated with one specific stimulus category within the session (either flanged or unflanged stimuli). Because there was only one dot on the screen (either green or red), they were “forced” to select this one. After their response, they would be presented with a stimulus from the corresponding category for 4 s (820 × 1134 px) and receive a reward, followed by a 2 s inter-trial interval. In total, subjects had to pass 8 forced trials (4 green, 4 red) at the start of each session, in order to probe the association between dot colour and stimulus category within the session.

Hereafter, they were presented with 16 choice trials. The start and end of each choice trial were essentially the same as for the forced trials. However, instead of being presented with *one* coloured dot, subjects could now choose between the red dot and the green dot, thereby controlling the stimulus category on the screen. The dots were presented in a circular way, equidistant from the center of the screen and always located exactly opposite of each other. Note that this differs from the method that Watson et al.^22^ describe, who presented the choice dots always at the same location on the screen. However, we noticed during the familiarisation sessions that the orang-utans would show anticipatory responses because they would know the exact location where the dots would appear. Therefore, we chose to randomize the location of the choice dots in a circular way. Importantly, the coloured dots were always located at the same distance from the center of the screen, where subjects needed to tap to advance to the choice dots.

With regard to training, all individuals were already familiar with clicking dots for a reward. Therefore, we mainly had to familiarise them with the specific task (between July and October 2021). To this effect, all participating subjects fulfilled eight sessions. The first six sessions presented them with pictures of animals and flowers. Importantly, in these sessions we had not yet implemented the randomized location of the choice dots. They were presented on fixed locations, as in the original method^22^. Because we noticed that individuals would sometimes anticipate the appearance of the choice dots by clicking their location repeatedly before onset, we decided to run two final training sessions in which we implemented the randomised circular presentation described above. Subjects could only participate in the experimental sessions after participating in all eight of the familiarisation sessions. In total, six subjects fulfilled this criterion: all individuals except for the two flanged males.

In total, each subject participated in six experimental sessions between September and December 2021, depending on whether the subject already finished the familiarisation phase. In three of the sessions, flanged stimuli were associated with red dots, and in the three other sessions, flanged stimuli were associated with green dots. Subjects were presented with the sessions in blocks based on the colour-stimulus category association, so that they did not have to re-learn the association each session. Thus, three individuals started out with the three sessions where green was associated with flanged stimuli, while the three other individuals started out with the sessions where red was associated with flanged stimuli. Within the 3-session colour blocks, the order of the sessions was randomized between subjects.

### Statistical analysis

We performed all of the analyses in R statistics Version 4.2.2. To analyse the data, we used Bayesian mixed models. Bayesian analyses have gained in popularity over the past few years because they have a number of benefits compared to frequentist analyses^53,54^. While frequentist methods (e.g., p-value null-hypothesis testing^55^)inform us about the credibility of the data given a hypothesis, Bayesian methods inform us about the credibility of our parameter values given the data that we observed. This is reflected in the different interpretation of frequentist and Bayesian confidence intervals: The first is a range of values that contains the estimate in the long run, while the latter tells which parameter values are most credible based on the data^53,56^. Furthermore, Bayesian methods allow for the inclusion of prior expectations in the model, are less prone to Type I errors, and are more robust in small and noisy samples^54^. Altogether, these reasons make Bayesian methods a useful tool for data analysis.

All models were created in the Stan computational framework and accessed using the brms package^57,58^, version 2.18.5. All models were run with 4 chains and 6000 iterations, of which 1000 were warmup iterations. We checked model convergence by inspecting the trace plots, histograms of the posteriors, Gelman-Rubin diagnostics, and autocorrelation between iterations^59^. We found no divergences or excessive autocorrelation in any model. Furthermore, we used the package emmeans^60^ to obtain posterior draws for contrasts. Below, we discuss the specific statistical models for each experiment.

#### Dot-probe task

In line with previous studies^5,7,38,42^ we filtered the reaction times (RTs). First, we excluded slow reaction times, because they might reflect low motivation or distraction. Instead of opting for a fixed outlier criterion, we determined the upper limit per subject based on the median absolute deviation (MAD) in RT (i.e., RT = median + 2.5 × MAD; Leys et al., 2013). Second, we excluded reactions times < 200 ms, because they very likely represent anticipatory responses^61^. These unsuccessful trials were afterwards repeated in subject-specific repetition sessions. After the repetition of these unsuccessful trials, we applied the same filtering criteria.

For the flange size dot-probe, we collected 423 trials of which 96 were excluded based on the outlier criteria (22.69%). In the subject-specific repetition sessions that consisted of the unsuccessful trials based on our outlier criterion, we collected 105 trials, 28 of which were excluded based on the outlier criteria (26.67%). Thus, our final dataset for the flange size dot-probe contained 404 trials (Kawan: 133; Samboja: 131; Sandy: 140). For the symmetry dot-probe, we followed the same procedure. In total, we collected 474 trials, 102 of which were excluded based on the outlier criteria (21.61%). In the subject-specific repetition sessions that consisted of the unsuccessful trials based on our outlier criterion, we collected 108 trials, 32 of which were excluded (29.63%). Thus, our final dataset for the symmetry dot-probe contained 448 trials (Kawan: 152; Samboja: 142; Sandy: 154).

For both experiments, we created separate statistical models per subject. We chose to analyze our data at the individual level because of the low number of subjects that participated in this experiment. Given the fact that we had a relatively high number of trials per subject, it was possible to test for the presence of a within-subject effect separately for each subject. Previous work has suggested that this is a suitable approach in case of low subject numbers^62,63^.

To test whether the orang-utans had an attentional bias for large flanges, we fitted three Bayesian mixed models with a Student-*t* family. The Student-*t* family is ideal for robust linear models, as the model will be influenced less strongly by outliers. We specified mean-centered RT (in ms) as dependent variable, and Congruence (Congruent: probe behind large flange stimulus; Incongruent: probe behind small flange stimulus) as categorical independent variable. We added Probe location (Left/Right) as categorical independent variable to control for possible side biases in RT. Furthermore, we allowed the intercept to vary by Session, so that the statistical model accounted for variation in RT between sessions. We specified a Gaussian prior with *M* = 0 and *SD* = 5 for the Intercept of the model. For the independent variables, we specified regularizing Gaussian priors with *M* = 0 and *SD* = 10. For the *nu* parameter of the Student-*t* distribution, we specified a Gamma prior with *k* = 2 and θ = 0.1. For all variance parameters, we kept the default half Student’s t priors with 3 degrees of freedom. To test whether orang-utans had an attentional bias for symmetrical faces, we followed the exact same procedure. However, the predictor Congruence now refers to the symmetry of the depicted face (Congruent: probe behind symmetrical stimulus; Incongruent: probe behind original stimulus). We used sum-to-zero coding for all of our categorical independent variables.

#### Preference task

For 5 of the 6 subjects we had a complete dataset of 96 choice trials. Only for Kawan we missed 4 trials, because he left twice at the end of an experimental session. Thus, our final dataset consisted of 572 datapoints. Because we had a larger number of subjects in this experiment, we chose to analyze the data in one statistical model. To examine whether the orang-utans preferred seeing a picture of flanged males over unflanged males, we fitted a Bayesian logistic mixed model (Bernoulli family). We specified the binary choice (1 = flanged, 0 = unflanged) as dependent variable. The within-subject categorical variable Colour Flanged, which represent whether the flanged stimuli were associated with the red or the green dot, was added as an independent variable, together with the between-subject variable Order, which represented whether the individual first received the sessions in which the red dot was associated with the flanged stimuli or in which the green dot was associated with the flanged stimuli. To explore the effect of dot location on the screen on probability of selection, we extended the model by adding a continuous predictor that was zero-centered and reflected the location of the dot representing flanged stimuli relative to the vertical middle of the screen (range − 0.35–0.35, with negative values representing the higher portion of the screen).

With regard to the random effects, we allowed the intercept to vary by Subject and allowed the intercept of Session to vary within Subject. Furthermore, we allowed the slope for Colour Flanged to vary by Subject, to take into account potential treatment effects between subjects. We specified a Gaussian prior with *M* = 0 and *SD* = 0.5 for the Intercept and independent variables of the model. Note that these priors are specified on the logit scale. For all variance parameters, we kept the default half Student-*t* priors with 3 degrees of freedom.

To explore temporal clustering and dispersion in the choices of the orang-utans, we developed an R script based on^64^ that is essentially a Beta-Binomial model that can be used to assess independence of binary observations. We applied it to each of the sessions independently. The script first counts the number of switches between selected categories within the session (variable *T*). Second, we specified a Beta(10, 10) prior on *θ*, the probability of selecting a flanged male stimulus, emphasizing a relatively strong expectation of 50/50 selection of flanged and unflanged stimuli. Third, we obtained a posterior for *θ* by updating the Beta(10, 10) prior based on the choices from the session. Fourth, we simulated 10,000 binary series of the same length as the session, based on sampling from the posterior distribution of *θ*. Note that the binary series consisted of independent samples. Fifth, based on these simulations, we counted the number of switches *T* in each independent series, and obtained a distribution of *T* under the assumption of independence. This allowed us to compare the observed *T* within the sessions with the expected *T* under the assumption of independence. Consecutively, we checked whether the observed *T* fell outside of the 95% Highest Density Interval of the expected *T*, and we calculated the proportion of expected *T*-samples that was either similar or higher, or similar or lower than the observed *T*. With regard to the interpretation, an observed *T* that is low compared to the distribution of expected *T* reflects fewer switches in a session than expected under the assumption of independence, hence temporal clustering of choices. An observed *T* that is high compared to the distribution of expected *T* reflects more switches in a session than expected under the assumption of independence, hence temporal dispersion of choices.

#### Effect size indices

The effect size indices that we report are based on the posterior distributions of each statistical model. We report multiple quantitative measures to describe the effects. First, we report the median estimate (*b* or *OR*), and median absolute deviation of the estimate between square brackets. Second, we report an 89% highest density interval of the estimate (89% CrI). We have chosen 89% instead of the conventional 95% to reduce the likelihood that the credible intervals are interpreted as strict hypothesis tests^56^. Instead, the main goal of the credible intervals is to communicate the shape of the posterior distributions. Third, we report the probability of direction (*pd*), i.e., the probability of a parameter being strictly positive or negative, which varies between 50 and 100%^54^.

### Ethics

This study employed only non-invasive methods and animals were never harmed or punished in any way during the study. Participation was completely voluntary, animals were tested in a social setting, and animals were never deprived of food or water. The care and housing of the orangutans was adherent to the guidelines of the EAZA Ex-situ Program (EEP). Furthermore, our research complied with the ASAB guidelines^65^ and the ARRIVE guidelines^66^, was carried out in accordance with the national regulations, and was approved by the zoological management of Apenheul Primate Park (Apeldoorn, The Netherlands).

## Results

### Dot-probe

#### Flange size

**Figure 3:**
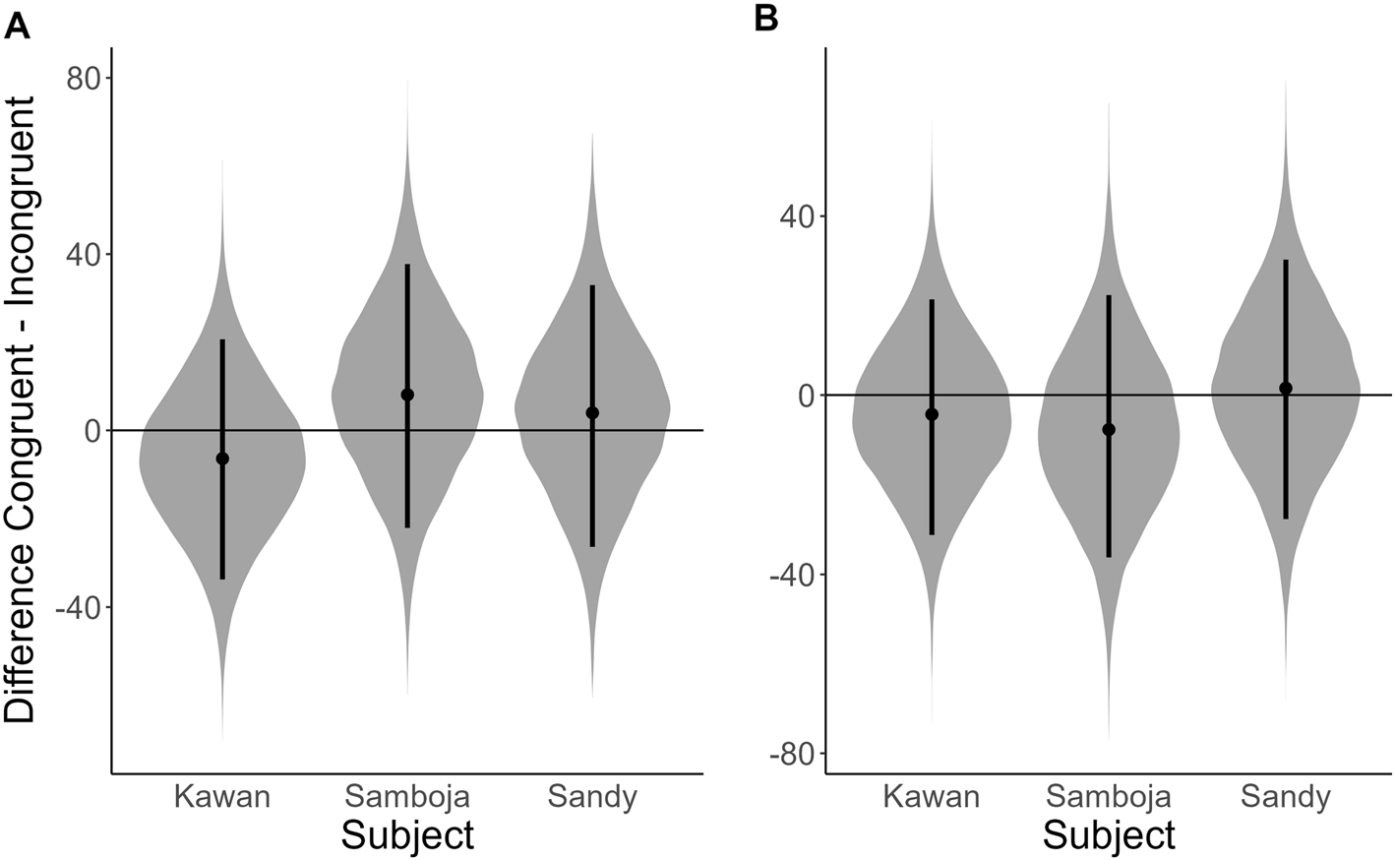
Posterior predictions of the difference in RT between trials where the probe replaced (**A**) the stimulus with larger flanges (Congruent) and trials where the probe replaced the stimulus with smaller flanges (Incongruent), or (**B**) replaced the stimulus with symmetrized face (Congruent) and trials where the probe replaced the stimulus with original face (Incongruent). Values under the horizontal null-line mean that the subject was predicted to respond faster to congruent than incongruent trials.

In the flange size dot-probe, we found no attentional bias for larger flanges in any of the three participating orang-utans (Fig. 3A; Supplementary Table 2); whether the probe replaced the large or small flange picture had no robust effect on the RT of Kawan (*b*_congruent_ = -3.28 [8.50], 89% CrI [− 16.66; 10.49], *pd* = 0.65), Samboja (*b*_congruent_ = 3.90 [9.38], 89% CrI [− 10.68; 19.25], *pd* = 0.66), and Sandy (*b*_congruent_ = 2.08 [9.13], 89% CrI [− 13.05; 16.52], *pd* = 0.59). We also found no robust effect of probe location (left/right) on RT, indicating that Kawan (*b*_left_ = 5.08 [8.66], 89% CrI [− 9.05; 18.41], *pd* = 0.72), Samboja (*b*_left_ = − 6.61 [9.41], 89% CrI [− 21.82; 8.60], *pd* = 0.76), and Sandy (*b*_left_ = 7.58 [9.30], 89% CrI [− 7.33; 22.12], *pd* = 0.80) did not have a side bias.

Because we applied a proportional transformation to our stimuli, the absolute width difference between the stimuli was not similar for all stimulus combinations. Therefore, we ran additional sensitivity analyses that explored whether the difference in RT between congruent and incongruent trials varied over the absolute width difference of the stimuli. These analyses are reported in the Supplementary Materials (Supplementary Table 3; Supplementary Fig. 1). We found no indications that the orang-utans did show a faster response to congruent trials at specific width differences. This suggests that our null results are at least not driven by differential responses to stimuli on the extremes of the width spectrum.

#### Symmetry

In the symmetry dot-probe, we found no attentional bias for symmetrical faces in any of the three participating orang-utans (Fig. 3B; Supplementary Table 4); whether the probe replaced the large or small flange picture had no robust effect on the RT of Kawan (*b*_congruent_ = -3.28 [8.50], 89% CrI [− 16.66; 10.49], *pd* = 0.65), Samboja (*b*_congruent_ = 3.90 [9.38], 89% CrI [-10.68; 19.25], *pd* = 0.66), and Sandy (*b*_congruent_ = 2.08 [9.13], 89% CrI [− 13.05; 16.52], *pd* = 0.59). Similar to the flange size experiment, we found no robust effect of probe location (left/right) on RT, indicating that Kawan (*b*_left_ = 5.08 [8.66], 89% CrI [− 9.05; 18.41], *pd* = 0.72), Samboja (*b*_left_ = − 6.61 [9.41], 89% CrI [− 21.82; 8.60], *pd* = 0.76), and Sandy (*b*_left_ = 7.58 [9.30], 89% CrI [− 7.33; 22.12], *pd* = 0.80) did not have a side bias.

### Preference task

**Figure 4:**
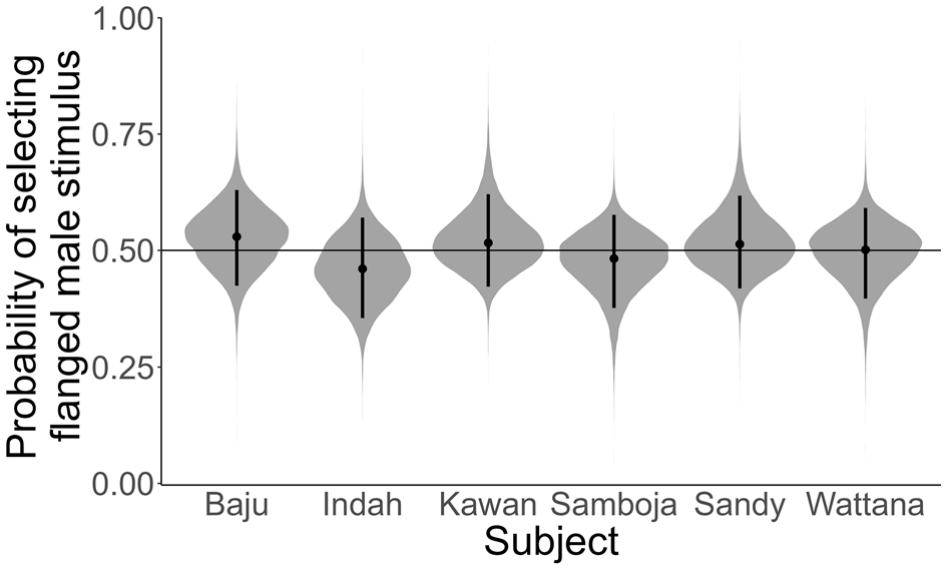
Posterior predictions of the probability of selecting the flanged male stimulus per subject. The horizontal line indicates chance level.

In the preference test (Supplementary Table 5), we found that the orang-utans chose stimuli of flanged and unflanged males exactly at chance level (OR_Intercept_ = 1.00 [0.13], 89%CrI [0.78; 1.25], *pd* = 0.52). Thus, they did not seem to prefer looking at stimuli of flanged males. This was the case for all individuals (Fig. 4): Baju (OR_Intercept_ = 1.13 [0.30], 89%CrI [0.66; 1.61], *pd* = 0.67), Indah (OR_Intercept_ = 0.85 [0.24]], 89%CrI [0.51; 1.27], *pd* = 0.72), Kawan (OR_Intercept_ = 1.07 [0.26], 89%CrI [0.68; 1.56], *pd* = 0.61), Samboja (OR_Intercept_ = 0.93 [0.23], 89%CrI [0.57; 1.31], *pd* = 0.55), Sandy (OR_Intercept_ = 1.06 [0.25], 89%CrI [0.66; 1.54], *pd* = 0.59), and Wattana (OR_Intercept_ = 1.00 [0.13], 89%CrI [0.78; 1.25], *pd* = 0.52).

**Figure 5:**
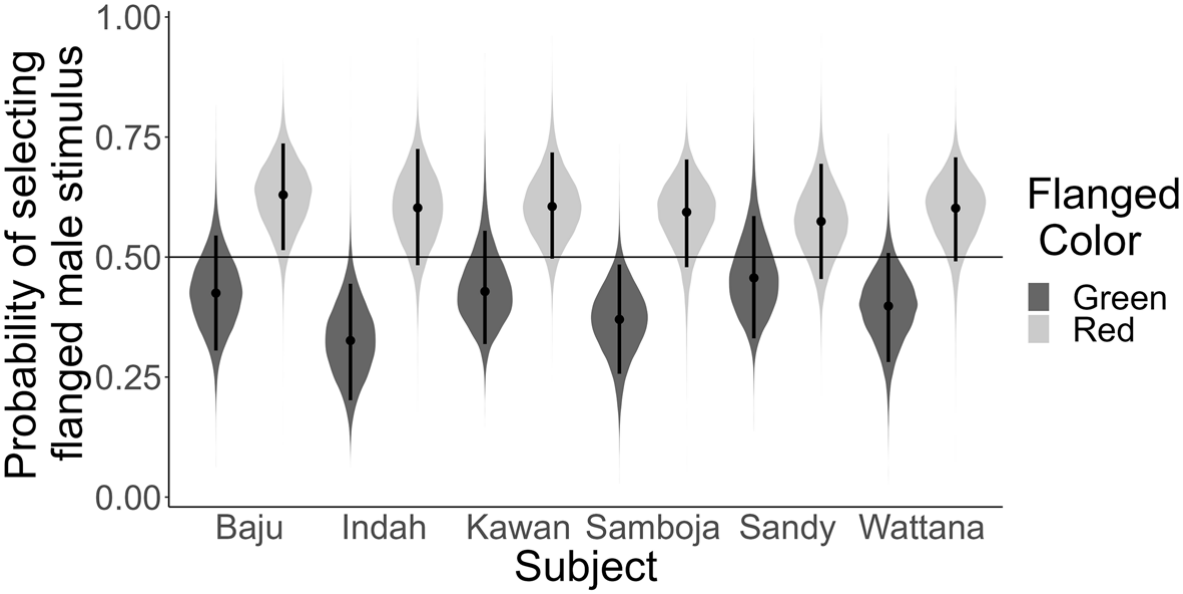
Posterior predictions of the probability of selecting the flanged male stimulus as a function of the colour associated with flanged male stimuli per subject. The horizontal line indicates chance level.

**Figure 6:**
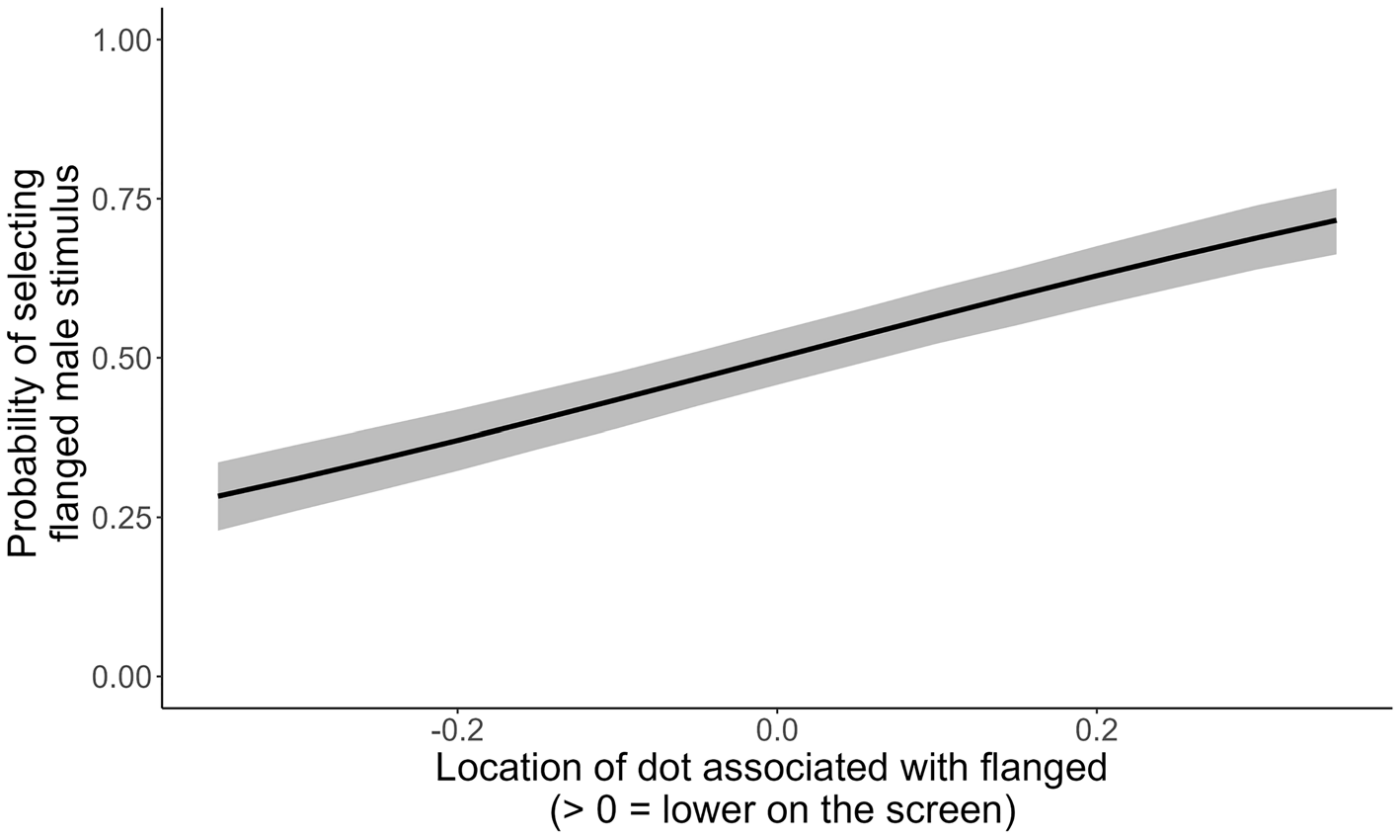
Posterior predictions of the probability of selecting the flanged male stimulus as a function of the vertical position of the dot representing the flanged male on the screen. Negative values indicate that the dot associated with the flanged male stimulus was positioned in the higher portion on the screen, while positive values indicate the lower portion of the screen.

The between-subject effect of Order did not have a robust effect on the preference of the individuals (OR_FlangedRedFirst_ = 0.88 [0.11], 89%CrI [0.69; 1.07], *pd* = 0.84). However, the colour of the dot that was associated with flanged males did have an influence on the preference: the orang-utans were more likely to select the flanged male stimulus if these were associated with the red dot (OR_Green_ = 0.67 [0.08], 89%CrI [0.54; 0.83], *pd* = 0.99), indicating a preference for the colour red (Fig. 5). Furthermore, we found very strong evidence for the notion that orang-utans made energy-efficient choices (Supplementary Table 6; Fig. 6): they were more likely to select the flanged stimulus when the dot associated with it was presented in the lower portion of the screen (OR_Height_ = 17.01 [5.06], 89%CrI [9.42; 25.64], *pd* = 1.00).

In addition, we explored whether individuals showed temporal clustering in their choices by selecting the same category multiple times in a row. To this effect, we compared the number of switches between categories for every session to a dataset consisting of the number of switches that one would expect under the assumption of independence. We found no evidence for temporal clustering (fewer switches than expected) or temporal dispersal (more switches than expected) in any of the sessions, indicating that previous choices did not influence choices in the next trial.

## Discussion

Even though face perception in primates has been studied extensively, the interplay between facial traits relevant to mate choice and cognition has received relatively little attention, especially in great apes. Therefore, the aim of this study was to investigate whether zoo-housed Bornean orang-utans (*Pongo pygmaeus*) have cognitive biases for males with fully developed secondary sexual traits (flanged males) or males with more symmetrical faces. Across two experiments, measuring either immediate attention bias or choice bias, we found no evidence of cognitive biases towards facial traits that might be relevant for mate choice. This lack of biases was consistent between all participating individuals. Furthermore, we did not find evidence for either temporal clustering or dispersion in the preference test: orang-utans did not seem to alter their choices based on their response in previous trials. However, we did find evidence of (i) a robust colour bias and (ii) an energy conservation strategy in the preference test. Below, we discuss our results in the context of primate literature and orang-utan ecology and consider methodological limitations.

Contrary to our hypotheses, we found no evidence for immediate attentional biases towards either large flanges or symmetrical faces in the dot-probe paradigm, while we expected a bias towards larger flanges and more symmetrical faces. With regard to flanges, previous research has shown that orang-utans spend a substantial amount of time looking at flanges while scanning male faces^3^ and orang-utans also showed an attentional bias towards flanged males in an eye-tracking study^67^. Regarding symmetry, we recently reported a similar null result in humans in the exact same task^15^: human participants had no attentional bias towards symmetrical faces. While previous literature has often emphasised the importance of symmetry for mate choice^68,69^, recent literature has criticised this notion in humans on the basis that the link between symmetry and attractiveness seems overstated^32^ and the link between symmetry and health remains equivocal^33^. Thus, the results for facial symmetry are in accordance with recent null findings and theoretical debates in humans.

While a null result could indicate that orang-utans do not have an immediate attention bias towards larger flanges or symmetrical faces, there are relevant methodological limitations in our dot-probe study that warrant some reflection. First, specifically regarding the symmetry experiment, we presented artificial stimuli (symmetrized versions) paired with the original faces. Therefore, there was a risk that we investigated attention bias to manipulated versus unmanipulated images instead of symmetrical versus asymmetrical faces. It is difficult, however, to envision how this could have led to null results. If the orang-utans indeed showed a clear bias towards either category, this would be a convincing alternative explanation. Unfortunately, no studies have yet investigated whether orang-utans have an attentional bias towards unmanipulated or manipulated stimuli. However, recent studies in rhesus macaques have not found evidence that natural images are attended to in a different way than “uncanny” manipulated stimuli^70,71^. Nevertheless, future studies could consider employing morphing techniques^72^ to create manipulated versions of both symmetrical and asymmetrical faces. Such methods allow for symmetrizing the shape of the face without changing any other textural or structural parameters.

Moreover, it is possible that the manipulation we used, which involved presenting faces with slightly larger or smaller flanges, did not generate salient enough differences between the stimuli to produce robust variations in reaction times in an immediate attention task. Instead of presenting the orang-utans with pictures of different flanged and unflanged males, we wanted to present the same faces while varying only the size of the flanges. This is a common approach in such studies (e.g. in macaques^19^ & humans^73^) to keep the stimuli as controlled as possible*.* A more skeptical interpretation would be to question whether the orang-utans could even distinguish between the smaller and larger stimuli or between symmetrical and asymmetrical faces. Previous size discrimination studies showing that primates can distinguish objects that approximately differ 10% in volume^74^ and that chimpanzees are able to discriminate between dots that differ < 10% in size^75^. Given that our stimuli differed on average 15% in width and none being < 10%, we think it is unlikely that the orang-utans would not have been able to distinguish between the larger and smaller stimuli. The same applies to facial symmetry: previous studies have shown that different primate species are sensitive to variation in facial symmetry (rhesus macaques^24^ & capuchin monkeys, *Sapajus apella*^72^). To our knowledge, there are no studies investigating explicit categorizing of symmetrical and asymmetrical faces in primates. However, even if primates were not able to explicitly do so, this would not mean that their attention cannot be implicitly biased differentially by symmetrical and asymmetrical faces. Such contradictions between implicit and explicit cognition can also be found in attentional tasks with humans. For example, people may implicitly avoid attending to specific locations that often contain distractor images while at the same time not being able to explicitly indicate those locations^76^. Altogether, we deem it unlikely that the orang-utans were not able to discriminate between larger and smaller flanges or symmetrized and asymmetrical faces, while at the same time acknowledging that more extreme manipulations of the stimuli might have resulted in an attentional bias. However, this would mean that we would present the orang-utans with extremely unnatural stimuli, which would affect the ecological validity of our results.

Another important limitation is that the experimental paradigm that we used to study immediate attention, the dot-probe paradigm, has been subject to debate in humans due to its relatively poor reliability^77,78^. Similarly, some inconsistent results have been observed when applying this paradigm to primates. While the paradigm has successfully shed light on the influence of emotion information on cognition in various primate species^7,41,42,52,79^, inconsistencies persist. For example, we have recently shown that Bornean orang-utans do not seem to show the expected attentional bias towards emotions in the dot-probe task^38^. This raises the question of whether such a widely reported bias is genuinely absent in Bornean orang-utans or if the current paradigm fails to capture it adequately. One potential methodological reason for these inconsistencies is that the dot-probe paradigm relies on reaction times, which are inherently noisy^80^. Especially for species with relatively low levels of manual dexterity compared to humans, such as orang-utans^81^, reaction time might not be the most suitable dependent measure in cognitive tasks. Instead, more fine-scaled methods such as non-invasive eye-tracking could be considered to study attentional preferences in primates. These methods are relatively easy to implement in primates^82^, and provide a more direct measure of attention^83^. Correspondingly, we did find an immediate attention bias towards flanged males in an eye-tracking task (Roth et al., in prep.). This suggest that eye-tracking allows us to probe cognitive biases that are potentially too subtle to identify using reaction time tasks, at least in orang-utans.

In the preference task, we used a previously developed paradigm^22^ to test whether Bornean orang-utans would choose to be presented with flanged or unflanged stimuli. However, all individuals selected flanged and unflanged stimuli equally often. Our results are in contrast with the results that a previous study found in rhesus macaques^22^, who specifically selected stimuli depicting faces of high-ranking individuals or stimuli showing coloured perinea. While we made some minor adaptations to the original paradigm (longer stimulus presentation, no fixed dot locations to avoid anticipatory responses, no indirect comparison of stimulus categories), we do not consider it likely that these changes explain the null results. One potential explanation relies on the fact that both choices were rewarded equally, meaning that there was no incentive to choose one category over the other in principle. Because Bornean orang-utans are often confronted with long periods of fruit scarcity^48^, they might be especially sensitive to food reward. Potentially, the anticipation of reward during the trial was so salient for them that the means to get to the reward became relatively unimportant. This raises the question whether extrinsically rewarded touchscreen experiments like the one we used here are suitable to study Bornean orang-utan cognition.

We also found that individuals had a higher tendency to choose the flanged male stimulus when it was associated with a red-coloured dot instead of the green-coloured dot, despite the fact that the dots were similar in saturation. This preference for red may indicate a general sensory bias towards the colour red, which could be attributed to the evolutionary pressure on primates to select ripe fruits or young leaves^84^. This bias for red objects might extend beyond fruits, possibly explaining why the individuals in the study were more likely to select the red dot. However, previous reports present conflicting evidence regarding the colour bias in food preferences among orang-utans. While one report suggested a preference for red food in a juvenile orang-utan^85^, a more recent report did not find any colour bias^86^. It is important to note that both reports concern single-subject observations. A more comprehensive study in rhesus macaques demonstrated a bias towards red food items, but this bias did not extend to non-food objects^87^. In conclusion, we found evidence for the notion that orang-utans have a sensory bias towards red objects, although this seems to conflict somewhat with existing literature on colour biases in primates.

In addition, orang-utans were more likely to select the dot associated with flanged male stimuli if it was in the lower portion of the screen, potentially reflecting an energy conservation mechanism. Bornean orang-utans are extremely well-adapted to low fruit availability. This is reflected in their extremely low levels of energy expenditure^46^ and their energy-efficient locomotion style^88,89^. This inclination to conserve energy may also manifest in their behaviour during our experiment. In the preference tasks, the locations of the dots were randomized in a circular way between trials, with both dots appearing in exact opposite positions equidistant from the center of the screen. While this approach helped to avoid anticipatory clicking by the orang-utans, it did result in differential energy costs associated with the dots. Clicking the dot in the upper portion of the screen required them to lift their arm further compared to clicking the dot in the lower portion of the screen. Consequently, the orang-utans were more inclined to select the dot in the lower portion of the screen. It is important to acknowledge this limitation in our experimental design. Nevertheless, even after accounting for the vertical location of the dots, we found no bias for flanged or unflanged stimuli (Supplementary Table 6). Thus, the strong tendency of orang-utans to conserve as much energy as possible may influence their performance during cognitive tasks.

Future studies on orang-utan cognition should consider the aforementioned effects of colour and dot location on choices. These biases underscore the need for a biocentric approach to animal cognition, which takes into account a species' uniquely adapted perceptual system^90^. Interestingly, however, the notion that orang-utans try to conserve energy during cognitive tasks opens up intriguing avenues for further research. If orang-utans are so prone to conserve energy, it might be possible to exploit this tendency by presenting them with an effort task. Previous studies with primates have developed effort paradigms that are relatively easy to use. These paradigms allow individuals to control the presentation of stimuli by holding a button (i.e., exerting effort). For example, previous studies have used this approach to study preferences for different stimulus categories in Japanese macaques (*Macaca fuscata*), finding that they exerted more effort to see stimuli of monkeys^91^ or humans^92^. A similar design could be considered for orang-utans: given that energy conservation is such a core strategy for them, using an effort task may be an especially relevant method to induce their preferences for specific stimuli categories.

In conclusion, our findings from two experimental paradigms indicate no immediate attentional bias towards large flanges or symmetrical faces, nor a choice bias for flanged males. However, we did find a preference for the colour red in the preference task. Furthermore, individuals seemed to conserve energy during the preference task by picking the vertically lowest option on the touchscreen. Our results highlight the importance of taking species-typical characteristics into account when designing cognitive experiments. Future studies could leverage the energy-conserving nature of Bornean orang-utans by presenting them with effort tasks, where they need to exert effort to view stimuli. Such an approach may be fruitful to study social cognition, including its interplay with mate choice, in Bornean orang-utans.

### Supplementary Information

Supplementary Information.

## Data Availability

The datasets and materials generated and/or analysed during the current study are available via DataverseNL: 10.34894/BL87ES.
