# Supplementary material for: No immediate attentional bias towards or choice bias for male secondary sexual characteristics in Bornean orang-utans (Pongo pygmaeus)
Source: Sci Rep. 2024 May 27;14:12095. doi: 10.1038/s41598-024-62187-9 (PMC11130206; doi:10.1038/s41598-024-62187-9)
Supplement: Supplementary file 1 — Supplementary Information. [file 41598_2024_62187_MOESM1_ESM.docx]

No immediate attentional bias towards or choice bias for male secondary sexual characteristics in Bornean orang-utans (*Pongo pygmaeus*)

Tom S. Roth

Iliana Samara

Juan Olvido Perea-Garcia

Mariska E. Kret

*Scientific Reports*

Supplementary Materials

Supplementary methods

**Training protocol**

We trained the orang-utans in the 5 months prior to the data collection period. Our training protocol was based on protocols employed by Kret et al. (2016) to test bonobos (*Pan paniscus*) and Laméris et al. (2022) to test Bornean orang-utans (*Pongo pygmaeus*) on the dot-probe task. This training protocol consists of several steps. Because all individuals were previously already exposed to the touchscreen, they did not need any habituation tot the presence of the touchscreen setup. First, we presented a smaller black dot (200x200 pixels) that they could click for a reward. Second, we extended this by training them on clicking two dots: one centrally placed and one either on the left or right side of the screen, as in the dot-probe task. Third, we presented individuals with a training task where they first needed to click a centrally placed dot after which they were presented with a dot and a picture on opposite sides of the screen. Here, they would be rewarded for clicking the dot, but not the picture. We added this step to ensure that they would not respond to the pictures appearing on the screen, but to the dot instead. Fourth, we trained them on the full dot-probe experiment with task-irrelevant stimuli (animals, landscapes, flowers). When individuals reached at least 80% accuracy on two consecutive training days, we presented them with the next training task until they reached the full dot-probe version. Trials were considered correct and rewarded when the orangutans correctly touched the dot and the subsequent probe and when they were attending the task for the entire trial.

The orang-utans in Apenheul were housed in four sub-enclosures during the study period, two of which had access to the test setup. Due to regular rotation between enclosures, all individuals had access to the setup. We scheduled approximately two training days per week. Importantly, we did not separate the individuals from their social group if they were housed together with other individuals. Thus, individuals could be surrounded by conspecifics during training. Nevertheless, we trained them individually by either distracting the other individuals with food enrichment or pausing the session whenever another orangutan interrupted. Sessions were furthermore conducted using positive reinforcement training, using sunflower seeds as reward for each correct trial. Individuals participated in a maximum of four training sessions on a test day. Each training session consisted of 20 trials.

Two individuals (Samboja & Sandy) previously participated in a dot-probe task (Laméris et al., 2022). Therefore, we immediately presented them with the final version of the training task: the full dot-probe task with task-irrelevant stimuli. They immediately fulfilled the 80% accuracy. During the training period, we kept presenting them this version of the task on days that they had access to the test setup in order to keep them motivated to interact with the screen. For the two flanged males, we quickly found that they had motivational issues, as was the case during training for the previous dot-probe study. Kevin showed little to no interest in the touchscreen. While Amos passed the first training task by hitting the touchscreen with his fist, he lost interest in the screen after this. We think that their motivational issues may have had to do, at least partly, with their large hands which obscured their view of the screen. During the training period, Baju and Indah were still highly dependent on their mothers, and therefore we could not properly train them. Thus, training mainly focused on Kawan and Wattana.

**Supplementary Table 1** – Training process per individual. Per training task, we indicate the number of training days needed to reach the accuracy criterion and between brackets the total number of sessions. ‘x’ means that an individual did not pass this criterion or skipped it. See the ‘Notes’-column for further clarification.

| **Individual** | **Training task 1** | **Training task 2** | **Training task 3** | **Training task 4** | **Notes** |
| --- | --- | --- | --- | --- | --- |
| Amos | 2 (4) | x | x | x | Dropout due to lack of motivation. |
| Baju | 2 (2) | 2 (3) | x | x | Dependent infant. We tried to train Baju, but because he was still so young he was often displaced during training. Therefore, we eventually were not able to properly train him. He participated in a few sessions of task 3, but did not fulfill the criterion. |
| Indah | x | x | x | x | Dependent infant. Still clinging to her mother during the training sessions. Thus, we were not able to train her on the task. |
| Kawan | 5 (13) | 2 (5) | 3 (7) | 4 (12) | After Kawan passed all training tasks, he participated in 12 more training sessions over 5 training days, because we were still training other individuals. We did this to keep him motivated. |
| Kevin | x | x | x | x | Dropout due to lack of motivation. |
| Samboja | x | x | x | 4 (8) | Already participated in previous dot-probe experiment. After Samboja passed the fourth task, she participated in 11 more training sessions over 5 training days, because we were still training other individuals. We did this to keep her motivated. |
| Sandy | x | x | x | 2 (4) | Already participated in previous dot-probe experiment. After Sandy passed the fourth task, she participated in 12 more training sessions over 4 training days, because we were still training other individuals. We did this to keep her motivated. |
| Wattana | 3 (12) | 2 (4) | x | x | Wattana did not manage to pass the third training criterion. In 9 sessions spread out over 4 training days, she never exceeded 70% accuracy. Hereafter, she lost interest in participating, also because Kawan showed a strong tendency to monopolize the setup after he successfully learned the task. Therefore, we decided not to continue the training process. |

**Supplementary Table 2** - Model outputs for the Flange Size dot-probe.

|  | **Kawan** | | **Samboja** | | **Sandy** | |
| --- | --- | --- | --- | --- | --- | --- |
| *Predictors* | *Estimates* | *CI (95%)* | *Estimates* | *CI (95%)* | *Estimates* | *CI (95%)* |
| Intercept | -0.23 | -9.95 – 9.36 | -0.95 | -10.69 – 8.64 | -0.14 | -9.80 – 9.68 |
| Congruence[LargeFlanges] | -3.28 | -19.79 – 13.37 | 3.90 | -14.35 – 22.15 | 2.08 | -15.99 – 19.99 |
| ProbeLocation[Left] | 5.08 | -11.74 – 21.90 | -6.61 | -25.43 – 12.14 | 7.58 | -10.45 – 25.68 |
| **Random Effects** | | | | | | |
| σ^2^ | 178.89 | | 301.29 | | 258.61 | |
| τ_00 Session_ | 960.64 | | 1735.80 | | 2012.05 | |
| Observations | 133 | | 131 | | 140 | |
| *Note: all categorical independent variables were sum-to-zero coded.* | | | | | | |
|  | | | | | | |

**Supplementary Table 3** - Model outputs for the Flange Size dot-probe with the interaction between difference in width (as a smooth predictor) and congruence.

|  | **Kawan** | | **Samboja** | | **Sandy** | |
| --- | --- | --- | --- | --- | --- | --- |
| *Predictors* | *Estimates* | *CI (95%)* | *Estimates* | *CI (95%)* | *Estimates* | *CI (95%)* |
| Intercept | -0.18 | -9.78 – 9.45 | -0.96 | -10.53 – 8.81 | -0.17 | -9.85 – 9.64 |
| Congruence[LargeFlanges] | -2.87 | -19.46 – 13.60 | 4.00 | -14.68 – 22.22 | 2.19 | -15.60 – 20.03 |
| ProbeLocation[Left] | 5.30 | -11.38 – 21.92 | -6.56 | -25.43 – 12.10 | 7.20 | -10.94 – 25.47 |
| bs_difference:congruence[LargeFlanges] | 0.27 | -19.39 – 20.01 | -0.09 | -19.73 – 19.49 | -0.38 | -20.00 – 19.13 |
| bs_difference:congruence[SmallFlanges] | -0.35 | -19.82 – 19.50 | 0.19 | -19.40 – 19.76 | 0.17 | -19.22 – 19.58 |
| sds_difference:congruence[LargeFlanges] | 148.45 | 19.11 – 429.53 | 100.45 | 4.61 – 481.97 | 120.85 | 5.28 – 533.32 |
| sds_difference:congruence[SmallFlanges] | 115.34 | 5.29 – 486.42 | 114.08 | 4.78 – 542.77 | 141.67 | 6.62 – 558.61 |
| **Random Effects** | | | | | | |
| σ^2^ | 173.44 | | 302.25 | | 257.63 | |
| τ_00 Session_ | 967.21 | | 1797.76 | | 1720.59 | |
| Observations | 133 | | 131 | | 140 | |
| *Note: all categorical independent variables were sum-to-zero coded.* | | | | | | |


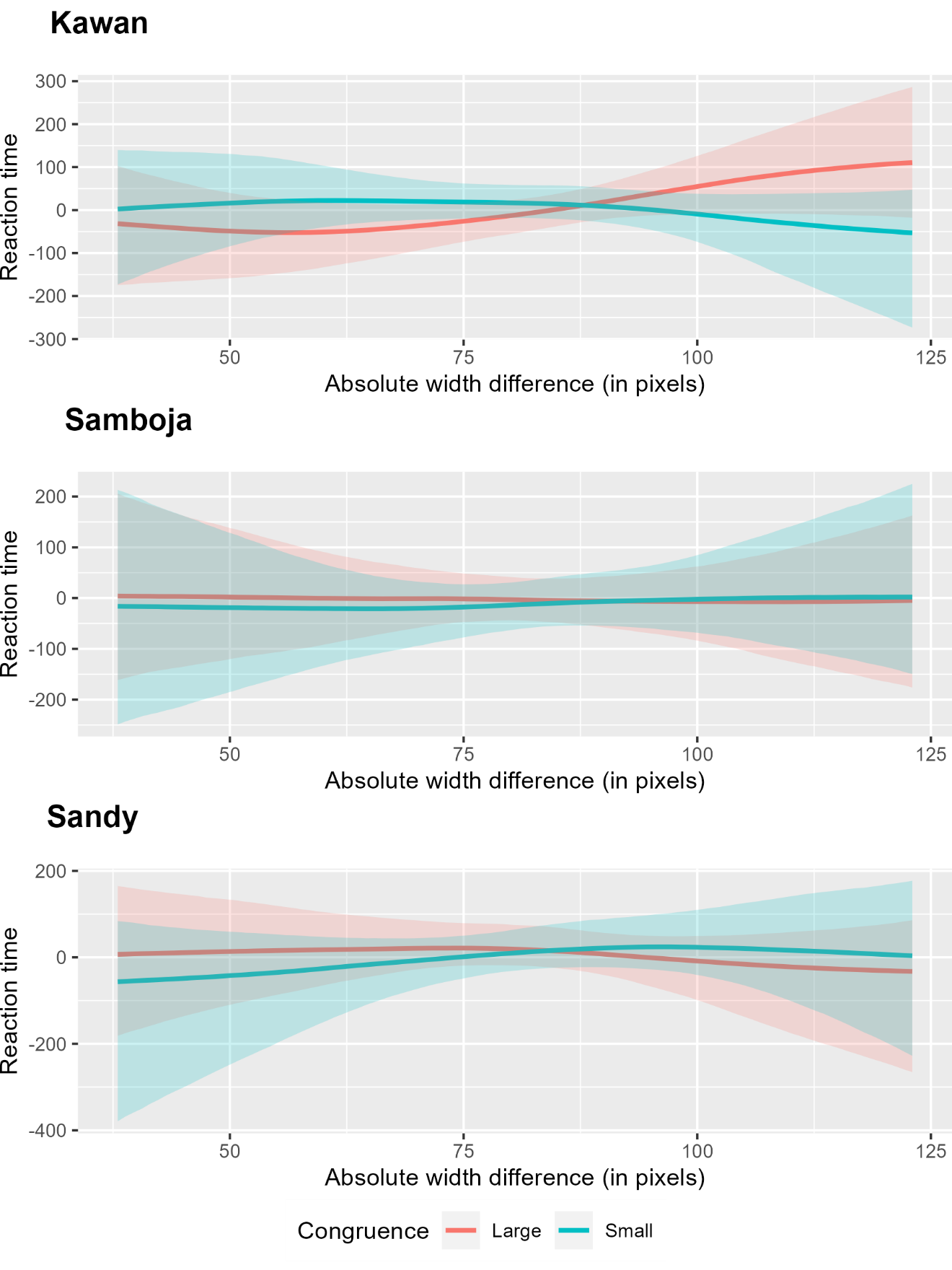


**Supplementary Figure 1** – Results of the interaction between width difference (smooth term) and congruence. We wanted to test whether there are specific width differences at which the orang-utans did respond faster at congruent that incongruent pictures. This could have indicated that some of our stimuli were not ecologically valid while others were. However, we see little variation in RTs to congruent and incongruent trials over the stimulus width. Only Kawan seems to show some variation, although the error margins still overlap considerably.

**Supplementary Table 4** - Model outputs for the Symmetry dot-probe.

|  | **Kawan** | | **Samboja** | | **Sandy** | |
| --- | --- | --- | --- | --- | --- | --- |
| *Predictors* | *Estimates* | *CI (95%)* | *Estimates* | *CI (95%)* | *Estimates* | *CI (95%)* |
| Intercept | -0.67 | -10.29 – 8.90 | -0.13 | -9.87 – 9.54 | -0.50 | -10.16 – 9.31 |
| Congruence[Symmetrical] | -2.10 | -18.13 – 13.99 | -3.82 | -22.22 – 14.26 | 0.81 | -17.21 – 18.90 |
| ProbeLocation[Left] | 12.09 | -4.24 – 28.09 | -7.11 | -24.98 – 10.94 | -1.11 | -19.08 – 16.78 |
| **Random Effects** | | | | | | |
| σ^2^ | 169.60 | | 276.77 | | 264.60 | |
| τ_00 Session_ | 2962.89 | | 3146.86 | | 4452.39 | |
| Observations | 152 | | 142 | | 154 | |
| *Note: all categorical independent variables were sum-to-zero coded.* | | | | | | |

**Supplementary Table 5** - Model output for the Flange Size preference test.

|  | **Preference** | |
| --- | --- | --- |
| *Predictors* | *Odds Ratios* | *CI (95%)* |
| Intercept | 1.00 | 0.73 – 1.38 |
| Color Flanged[Green] | 0.67 | 0.52 – 0.91 |
| Order[FlangedRedFirst] | 0.88 | 0.67 – 1.18 |
| **Random Effects** | | |
| τ_00_ _Subject_ | 0.11 | |
| τ_00_ _Subject:Session_ | 0.01 | |
| τ_11_ _Subject:Color Flanged_ | 0.09 | |
| N _SubjectName_ | 6 | |
| Observations | 570 | |
| *Note: all categorical independent variables were sum-to-zero coded.* | | |

**Supplementary Table 6** - Model output for the Flange Size preference test, including vertical location.

|  | **Preference** | |
| --- | --- | --- |
| *Predictors* | *Odds Ratios* | *CI (95%)* |
| Intercept | 1.00 | 0.74 – 1.35 |
| Color Flanged[Green] | 0.65 | 0.49 – 0.90 |
| Order[FlangedRedFirst] | 0.86 | 0.65 – 1.15 |
| Vertical_Location | 16.97 | 9.35 – 30.66 |
| **Random Effects** | | |
| τ_00_ _Subject_ | 0.27 | |
| τ_00_ _Subject:Session_ | 0.13 | |
| τ_11_ _Subject:Color Flanged_ | 0.26 | |
| N _SubjectName_ | 6 | |
| Observations | 570 | |
| *Note: all categorical independent variables were sum-to-zero coded. Continuous independent variable Vertical_Location was centralized around 0.5.* | | |
